# Tumor Necrosis Factor-α Variations in Patients With Major Depressive Disorder Before and After Antidepressant Treatment

**DOI:** 10.3389/fpsyt.2020.518837

**Published:** 2020-12-07

**Authors:** Lin Yao, LiHong Pan, Min Qian, Wei Sun, ChunHong Gu, LiangHu Chen, XiaoChen Tang, YeGang Hu, LiHua Xu, YanYan Wei, Li Hui, XiaoHua Liu, JiJun Wang, TianHong Zhang

**Affiliations:** ^1^Nanhui Mental Health Center, Pudong New Area, Shanghai, China; ^2^Department of Neurosurgery, Pu Nan Hospital, Shanghai, China; ^3^Shanghai Mental Health Center, Shanghai Jiaotong University School of Medicine, Shanghai Key Laboratory of Psychotic Disorders, Shanghai, China; ^4^Institute of Mental Health, Suzhou Guangji Hospital, The Affiliated Guangji Hospital of Soochow University, Soochow University, Shanghai, China; ^5^Bio-X Institutes, Key Laboratory for the Genetics of Developmental and Neuropsychiatric Disorders (Ministry of Education), Shanghai, China; ^6^Brain Science and Technology Research Center, Shanghai Jiao Tong University, Shanghai, China

**Keywords:** remission, serum, antidepressant, major depressive disorder, tumor necrosis factor-α

## Abstract

Tumor necrosis factor-α (TNF-α) had been identified as a key pro-inflammatory cytokine in the pathophysiology of major depressive disorder (MDD) and the mechanism of antidepressant treatment. The primary aim of the present study was to examine the serum TNF-α levels in Chinese inpatients with MDD during the acute phase and to explore the changes in TNF-α levels after effective clinical treatment. Fifty-seven consecutive inpatients with MDD and 30 healthy controls were recruited. The serum TNF-α levels were detected using ELISA. Symptoms of depression were evaluated using the 24-item Hamilton Rating Scale for Depression (HAM-D-24). TNF-α levels and HAM-D-24 scores were assessed at baseline and after 2 and 12 weeks of follow-up. The serum TNF-α levels were higher in the MDD group than in the control group. After 2 and 12 weeks of antidepressant treatment, there were significant improvements in the patients' symptoms and significant decreases in the TNF-α levels. The baseline TNF-α levels significantly correlated with the decreased HAM-D-24 scores, particularly for the depressive symptoms of anxiety/somatization and weight loss. The present findings indicate that depression is accompanied by activation of TNF-α, which also has a predictive value for the antidepressant treatment response in patients with MDD.

## Introduction

Major depressive disorder (MDD) has emerged as one of the most common psychiatric disorders (1). Extensive research efforts have been made to improve the understanding of its molecular pathophysiology. Although several mechanisms leading to MDD are possible, the inflammatory hypothesis is currently one of the widely accepted theories, also called the “cytokine” theory of depression (2). The inflammatory cytokine system is activated in several somatic diseases that share a number of common symptoms with MDD, such as tiredness. This implies that inflammatory cytokines may play a key role in the development of depression (3). This theory also well-explained some phenomena of psycho-neuroimmunological dysfunction in patients with MDD, who have an abnormal peripheral immune system (4). It has been established that inflammatory cytokines can induce immune stimulation, which induces depression-like signs and symptoms, and these links have been supported by experimental and clinical evidences (5, 6).

Tumor necrosis factor-α (TNF-α) is one of the essential pro-inflammatory cytokines that has received much attention because of its ability to cause inflammation and apoptotic cell death and to mediate the release of a variety of cytokines (7, 8). Two mechanisms may link TNF-α to the pathophysiology of MDD. First, pro-inflammatory cytokines such as TNF-α may regulate the neuronal serotonin transporter activity and stimulate serotonin uptake (9). Second, pro-inflammatory cytokines activate the tryptophan- and serotonin-degrading enzyme indolamine-2,3-dioxygenase, resulting in a reduced availability of serotonin in depression (10). Although from the mechanism point of view it has been postulated that the increased production of TNF-α might play a causative role in MDD, the levels of TNF-α have been shown to be quite varied among patients with MDD across many clinical investigations. Increased (11, 12), unchanged (13), and decreased (14) levels of TNF-α have been reported in depression. One of the possible explanations for this inconsistency might be that different patterns of TNF-α expression may appear in depressive episodes of different types.

Changes in the TNF-α system are involved not only in the development of MDD but also in mediating the response to antidepressant treatment. Two recent meta-analysis studies have found TNF-α to be decreased in MDD patients after antidepressant treatment, especially for those who are well-responded in the selective serotonin reuptake inhibitor (SSRI) treatment. However, due to the limited number of MDD cohort studies available, and the fact that the MDD sample was highly heterogeneous with respect to symptom severity and treatment response, the association between changes in TNF-α levels and response to antidepressant treatment in MDD was not clear.

Changes in the TNF-α system were involved not only in the development of MDD but also in mediating the response to antidepressant treatment. Two recent meta-analysis studies (15, 16) have found TNF-α to be decreased in MDD patients after antidepressant treatment, especially for those who are well-responded in the antidepressant treatment, but not for non-responders. However, due to the limited number of MDD cohort studies available (17–20), and the fact that the MDD sample was highly heterogeneous with respect to symptom severity and treatment response, the association between changes in TNF-α levels and response to antidepressant treatment in MDD was not clear. In addition, most studies (18–20) only measured the levels of TNF-α before and after treatment, and the treatment duration in those studies was varied, so it was impossible to observe the dynamic changes of TNF-α levels during the treatment process. Therefore, the present study explored by setting up multiple follow-up points to examine the dynamic changes in the serum TNF-α levels in a fairly homogeneous group of MDD patients treated with antidepressants in clinical settings. The primary aim of the current study was to examine the difference in the serum TNF-α levels in Chinese inpatients with MDD in the acute phase with healthy controls and, more importantly, to explore the changes in TNF-α levels and their predicting value for effective antidepressant treatment.

## Methods

### Overview

The current study included in patients with MDD from the Shanghai Nanhui Mental Health Center, the largest mental health service in Shanghai Nanhui district. The Research Ethics Committees of the Shanghai Pudong Nanhui Mental Health Center approved the study in 2018. The study was conducted in accordance with the tenets of the Declaration of Helsinki. A key element of this study was that all patients were drug-free (at least 2 weeks) prior to admission to the hospital.

### Sample and Study Design

All participants provided written informed consent at the recruitment stage of the study. A total of 87 subjects were included, 57 patients with MDD (MDD group) and 30 healthy controls (HC group), aged 29–82 years. The inclusion criteria for the patients with MDD were as follows: (1) diagnosis of MDD according to the criteria defined in the Diagnostic and Statistical Manual of Mental Disorders, fourth edition (DSM-IV) (21)—no additional diagnostic tool was applied—(2) an acute exacerbation of the symptoms of depression; and (3) a baseline score of at least 14 points on the 24-item Hamilton Rating Scale for Depression (HAM-D-24) (22). Each patient with MDD was asked to spend 1 week as an inpatient and undergo a clinical assessment and screening procedures (including physical examination, electrocardiography, and clinical laboratory investigations) before receiving antidepressant treatment. Other inclusion criteria were taking only one type of antidepressant during the study and willingness and ability to complete the 12-week follow-up assessment.

The exclusion criteria were as follows: (1) medical illness, such as chronic diseases, including diabetes, and any infectious disease in the month prior to enrollment; (2) pregnancy; (3) substance abuse; and (4) a history of use of relevant medications, such as antibiotics and antioxidants, within the previous 4 weeks. The physical examinations and routine standard laboratory tests for inpatients helped to exclude the subjects with medical comorbidities and concomitant medication use. At baseline, 30 healthy controls, volunteers from the community, were recruited. After they provided written consent, they were assessed with the Structured Clinical Interview for DSM-IV Axis I Disorders-Non-patient version (SCID-NP) to confirm that they had no past or present mental disorders. They underwent the same procedure of enrollment screening as did the patients with MDD, except the HAM-D-24 assessment.

### Clinical Assessment

The primary measure of the study was the HAM-D-24 scores. HAM-D-24 had been widely used in Chinese clinical practice and related studies for over 30 years. It was administered to the inpatients with MDD at baseline, and at 2 and 12 weeks follow-up. The 2-week follow-up time point was used to evaluate the short-term antidepressant effect, and the 12-week (average length of stay for inpatients in our hospital) follow-up time point was used to evaluate the mid-term antidepressant effect. HAM-D-24 assesses seven factors: (1) anxiety/somatization, (2) weight loss, (3) cognitive disturbance, (4) retardation, (5) diurnal variation, (6) sleep disorder, and (7) desperation. For example, the anxiety/somatization factor of the HAM-D-24 includes six items: anxiety-psychic, anxiety-somatic, somatic symptoms-gastrointestinal, somatic symptoms-general, hypochondriasis, and insight. All inpatients and outpatients were screened and assessed by a face-to-face interview by two senior psychiatrists. The agreement rate between the two psychiatrists was 0.8, expressed as a kappa value. The same clinical assessments, blood collection, and laboratory procedures were performed again at week 2 and 12 for all patients.

### Antidepressant Treatment

All the patients were taking one type of antidepressant during the follow-up: 40 of them were taking a SSRI (26 of them took escitalopram with a mean dose of 23.8 mg, 14 of them took paroxetine with a mean dose of 47.9 mg); 12, venlafaxine with mean dose of 187.5 mg; and 5, duloxetine with mean dose of 40 mg.

### Experimental Procedure

Five milliliters of blood was drawn by venipuncture into an anticoagulant-free tube between 6:00 a.m. and 7:00 a.m. after an overnight fast. The blood was centrifuged at 3,000 × g for 20 min at 4°C within 2 h of collection. Subsequently, the serum was separated and stored at −80°C until it was assayed.

Serum TNF-α levels were measured in duplicate using enzyme-linked immunosorbent assay (ELISA), with a commercial human ELISA kit (MULTISCIENCES(LIANKE) BIOTECH, CO., LTD), sensitivity 0.16 pg/ml (mean of 6 independent assays), in accordance with the manufacturer's instructions. The intra- and inter-assay coefficients of variation were <7 and 9%, respectively. No cross-reactivity was detected.

We average the duplicate readings for each standard and sample and subtract the average zero standard optical density. The data are linearized by plotting the log of the TNF-α concentrations vs. the log of the optical density (OD), and the best-fit line can be determined by regression analysis, the log of the TNF-α concentrations as horizontal axis, the log of the OD as the vertical axis. Then, we substitute the data into the standard curve and multiply by the dilution factor which is 5 to get the final concentration.

In all remaining study subjects, blood samples were tested for TNF-α concentration, with minimum level of 2.0 pg/mL and maximum of 25.2 pg/mL. There were no results that were undetected or below the sensitivity threshold. The concentration of TNF-α was expressed as pg/mL.

### Statistical Analysis

SPSS version 16.0 (IBM Corporation, Armonk, NY, USA) statistical software was used for all statistical computations. The sociodemographic and clinical characteristics of the participants were presented as descriptive statistics, such as percentages and mean scores. The daily antidepressant dose was converted to fluoxetine equivalent (23). A box-plot diagram was created using GraphPad Prism software to analyze the differences in the TNF-α levels between group HC and the baseline, week-2, and week-12 levels in group MDD. Independent *t* tests were conducted to measure group differences in continuous variables, and Chi-square statistics were used to examine categorical variables. The normality of the sample was confirmed by the Kolmogorov–Smirnov test, and the homogeneity was confirmed by the Levene test, after which the independent *t* tests were used to determine the group difference. Patients with MDD whose HAMD scores dropped below 8 were classified as the antidepressant-sensitive (AS) group, and others were classified as the antidepressant-insensitive (AIS) group (24). Two groups were compared by demographic and clinical characteristics and the TNF-α levels at baseline and follow-up points. The correlation between the baseline TNF-α levels and the percentage decrease in the HAM-D-24 scores after 2 and 12 weeks of antidepressant treatment was calculated using Spearman's non-parametric rank-correlation analysis. Subsequently, the relationship was explored via a partial correlation test for the seven factors of the HAM-D-24, controlling for the fluoxetine-equivalent dose of antidepressants. Finally, multivariate analysis with a linear regression model was performed to determine the regression coefficient (β) for the TNF-α levels, age, educational level, age at MDD onset, course of MDD, and equivalent antidepressant dosage for predicting HAM-D-24 score improvements at the week-2 and week-12 follow-up points. All reported results were two-tailed; significance was assumed at *p* < 0.05.

## Results

### Demographic and Clinical Characteristics

As shown in Table 1, the baseline age, education, and marriage data did not differ between the HC and MDD groups. The proportion of females was significantly higher in the MDD group than in the HC group. The majority of patients with MDD had significant reductions in the total HAM-D-24 scores at the 2 and 12-week follow-up evaluations. The clinical features, improvements, and antidepressant exposure in the MDD group are listed in Table 1.

**Table 1:** Comparison of the demographic, clinical, and treatment characteristics between healthy controls and patients with MDD.

**Variables**	**Healthy control**	**MDD**	**Comparisons**	
			***t/χ*^**2**^^a^**	***p***
Cases [*n*]	30	57	-	-
Age (years) [mean (SD)]	58.1 (15.1)	57.0 (10.6)	0.383	0.671
Age range (years)	29–82	29–77	*-*	-
Female [*n* (%)]	16 (53.3)	46 (80.7)	χ^2^ = 7.189	**0.007**
Education (years) [mean (SD)]	7.3 (3.4)	7.7 (3.0)	*t* = 0.521	0.604
Marriage_Single [*n* (%)]	1 (3.3)	6 (10.5)	χ^2^ = 1.998	0.573
Marriage_Married [*n* (%)]	25 (83.3)	42 (73.7)		
Marriage_Divorce [*n* (%)]	3 (10.0)	5 (8.8)		
Marriage_Widowed [*n* (%)]	1 (3.3)	4 (7.0)		
Family History [*n* (%)]	0 (0)	6 (10.5)	χ^2^ = 1.951	0.163
Age of MDD onset (years) [mean (SD)]	-	48.4 (14.2)	-	-
No. of MDD episode [mean (SD)]	-	2.6 (1.8)	-	-
No. of hospitalization [mean (SD)]	-	1.8 (1.5)	-	-
Course (months) [mean (SD)]	-	8.5 (8.6)	-	-
**Baseline HAM-D [mean (SD)]**
HAM-D total score [*n* (%)]	-	30.8 (4.8)	-	-
HAM-D factor-1: anxiety/somatization	-	8.5 (3.0)	-	-
HAM-D factor-2: weight	-	1.1 (0.9)	-	-
HAM-D factor-3: cognitive disturbance	-	3.6 (2.4)	-	-
HAM-D factor-4: retardation	-	2.1 (0.9)	-	-
HAM-D factor-5: diurnal variation	-	6.4 (1.6)	-	-
HAM-D factor-6: sleep disorder	-	4.9 (1.5)	-	-
HAM-D factor-7: desperation	-	4.2 (1.9)	-	-
**Antidepressants (mg per day) [mean (SD)]**				
Duloxetine	-	5 (8.8)	-	-
Escitalopram	-	26 (45.6)	-	-
Paroxetine	-	14 (24.6)	-	-
Venlafaxine	-	12 (21.1)	-	-
Fluoxetine-equivalent dose	-	75.8 (94.7)	-	-
**Week 2**			-	-
HAM-D total score [mean (SD)]	-	15.8 (3.2)	-	-
Δ1 HAM-D [mean (SD)]^b^	-	15.0 (3.1)	-	-
Δ1 HAM-D improvement rate (%) [mean (SD)]^c^	-	48.8 (6.5)	-	-
**Week 12**			-	-
HAM-D total score [mean (SD)]	-	7.5 (2.0)	-	-
Δ2 HAM-D [mean (SD)]^d^	-	23.3 (4.8)	-	-
Δ2 HAM-D improvement rate (%) [mean (SD)]^e^	-	75.2 (6.7)	-	-

a *t/χ^2^: t for t test, χ^2^ for kappa test*.

b *Δ1. HAM-D: baseline HAM-D—week 2 HAM-D*.

c *Δ1 HAM-D improvement rate (%): (baseline HAM-D—week 2 HAM-D)/baseline HAM-D*.

d *Δ2. HAM-D: baseline HAM-D—week 12 HAM-D*.

e *Δ2 HAM-D improvement rate (%): (baseline HAM-D—week 12 HAM-D)/baseline HAM-D*.

*Significant P values are bolded*.

### Differences in Serum TNF-α Levels Between HC and MDD Groups

**Figure 1:**
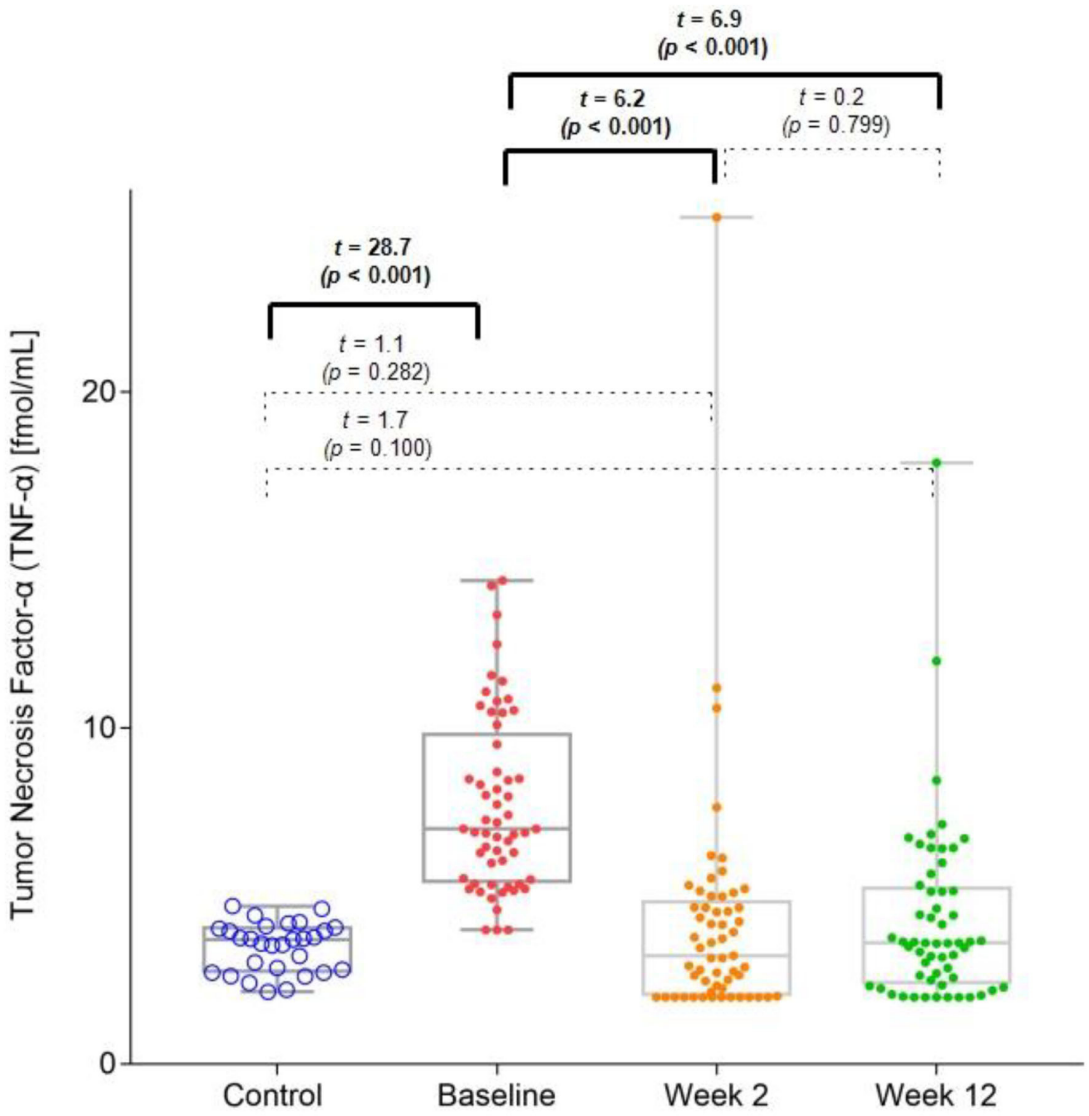
Differences in serum TNF-α levels among the different groups and follow-up points.

The MDD group had significantly higher baseline TNF-α levels (mean = 7.7 pg/ml [SD = 2.6]) than those of the HC group (mean = 3.5 pg/ml [SD = 0.7]). However, in both the 2-week (mean = 4.1 pg/ml [SD = 3.4]) and 12-week (mean = 4.3 pg/ml [SD = 2.7]) follow-up points of the trial, this difference between the groups was no longer significant (Figure 1). There was a significant decrease in the TNF-α levels from the baseline values at the 2 and 12-week follow-up points.

### Differences in Demographic and Clinical Characteristics, Serum TNF-α Levels Between as and AIS Groups

After 2 weeks of treatment with antidepressants, none of the patients' HAMD score dropped below 8. After 12 weeks of treatment, 42 of 57 (73.7%) patients with MDD were included in the AS group, and 15 patients were included in the AIS group. There were no significant differences in age, sex, age of MDD onset, course of disease, and baseline HAMD scores between the AS and AIS groups. The decreased HAMD score was higher in the AS group than in the AIS group (*p* = 0.044). The serum TNF-α levels at baseline in the AIS group were higher than those in the AS group (*p* = 0.015). However, there were no differences in the serum TNF-α levels at the 12-week follow-up points between the two groups (*p* = 0.218).

### Relationship Between Baseline TNF-α Levels and HAM-D-24 Score Improvement

**Figure 2:**
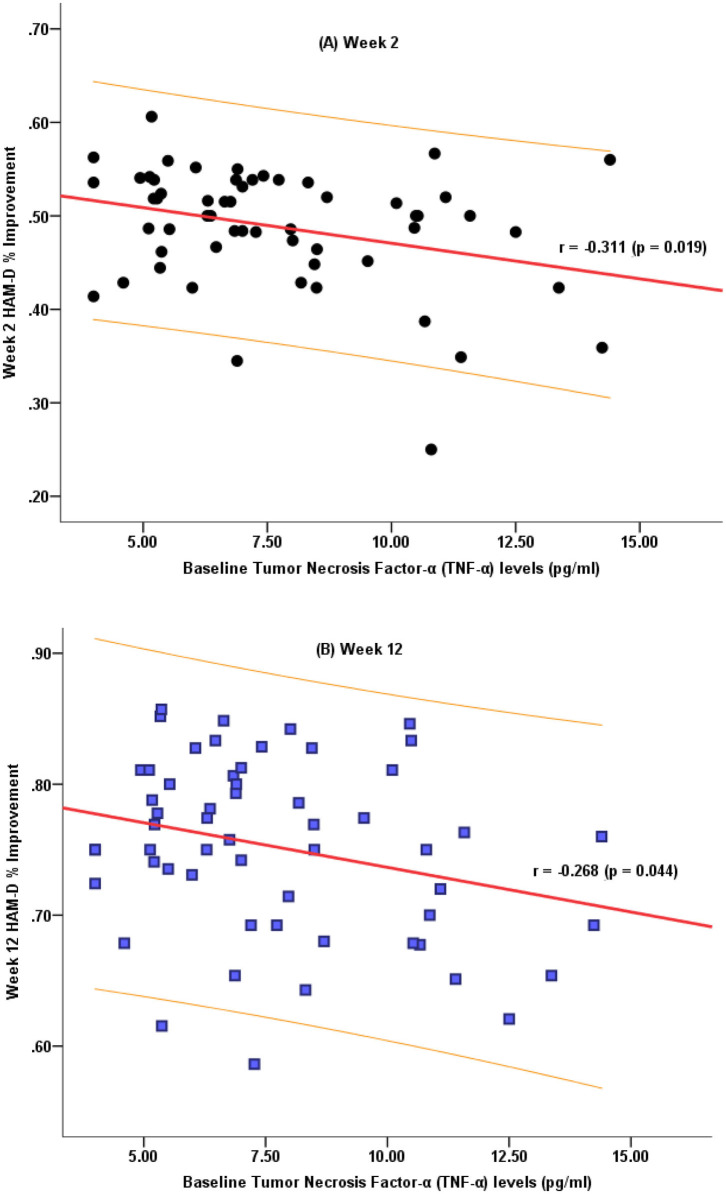
Correlation characteristics between the decrease in the HAM-D-24 total scores after 2 weeks **(A)** and 12 weeks **(B)** of treatment and the baseline TNF-α levels.

We further analyzed whether the baseline TNF-α levels correlated with the decreased HAM-D-24 scores. The percentage decrease in the HAM-D-24 scores after 2 and 12 weeks of antidepressant treatment were negatively correlated with the baseline TNF-α levels (Figure 2).

### Correlation Between the Baseline TNF-α Levels and Changes in the Depressive Subdomains After 2 and 12 Weeks of Treatment

At baseline, a significant correlation was found between the HAM-D-24 subdomains of anxiety/somatization and weight loss and the TNF-α levels. Table 2 highlights the observed significant correlation between the TNF-α levels and the weight loss subdomain score at week 2 and week 12 when controlling for fluoxetine-equivalent antidepressant dose.

**Table 2:** Correlation of the HAM-D-24 subdomains at baseline and after 2 and 12 weeks of treatment with the TNF-α levels.

**BASELINE**							
	**Anxiety/somatization**	**Weight loss**	**Cognitive disturbance**	**Retardation**	**Diurnal variation**	**Sleep disorder**	**Desperation**
*r*(TNF-α)	0.324	0.313	0.222	0.053	0.023	−0.150	0.020
*p*(TNF-α)	0.014	0.018	0.097	0.693	0.867	0.265	0.881
**WEEK 2 (PARTIAL CORRELATIONS WERE APPLIED BY CONTROL VARIABLE OF ANTIDEPRESSANT DOSAGE)**							
**Δ1(% Improvement)**	**Anxiety/somatization**	**Weight**	**Cognitive disturbance**	**Retardation**	**Diurnal variation**	**Sleep disorder**	**Desperation**
*r*(TNF-α)	0.005	0.290	0.106	0.174	−0.238	0.048	−0.196
*p*(TNF-α)	0.975	0.039	0.458	0.222	0.093	0.738	0.167
**WEEK 12 (PARTIAL CORRELATIONS WERE APPLIED BY CONTROL VARIABLE OF ANTIDEPRESSANT DOSAGE)**							
**Δ2(% Improvement)**	**Anxiety/somatization**	**Weight**	**Cognitive disturbance**	**Retardation**	**Diurnal variation**	**Sleep disorder**	**Desperation**
*r*(TNF-α)	−0.148	0.331	0.173	0.085	−0.019	0.028	0.046
*p*(TNF-α)	0.298	0.018	0.224	0.555	0.896	0.845	0.749

*Δ1 (% improvement): the percentage decrease in the HAM-D-24 factors from week 2 to baseline; Δ2 (% improvement): the percentage decrease in the HAM-D-24 factors from week 12 to baseline*.

### Prediction of the HAM-D-24 Score Improvement

Liner regression was used to evaluate the effect of the demographic and clinical variables and TNF-α levels on the HAM-D-24 score improvement rate at week 2, including age, educational level, age at MDD onset, course of MDD, equivalent antidepressant dosage, and baseline TNF-α levels. Consistently, only the baseline TNF-α level was found to significantly predict the week-2 HAM-D-24 score improvement rate in this model (β= −0.410, *t* = 2.487, *p* = 0.017). For the week-12 HAM-D-24 score improvement rate, two variables were added in the prediction model, the week-2 TNF-α levels and the improvement in the HAM-D-24 score at 2 weeks. Only the variable of improvement in the HAM-D-24 score at 2 weeks (β = 0.413, *t* = 2.970, *p* = 0.005) was found to be significant.

## Discussion

The main finding of the present study is that the serum TNF-α levels were significantly higher in patients with MDD than in healthy controls. Interestingly, after 2 and 12 weeks of antidepressant treatment, a significant improvement in the depressive symptoms with significant changes in the TNF-α levels were detected among the patients. The results of this study suggested that the serum TNF-α levels were sensitive to the depressive state, supporting the cytokine theory in the inflammatory hypothesis for the development of depression.

Several clinical studies have consistently reported that the serum TNF-α levels are increased in patients with depression (12, 25–27). Considering this, it is worthy to examine whether the TNF-α levels are associated with the antidepressant medication treatment and whether they could serve as a putative biomarker for the clinical response. However, owing to the heterogeneity of MDD and its treatments, the results of those studies are not consistent. Namely, TNF-α levels had been reported as increased (28), decreased (29), or unchanged (15) after effective antidepressant treatment. Since the antidepressants applied in current and other studies varied significantly, one possible reason for the inconsistent results is that different types of antidepressants may have different effects on TNF-α levels. For example, Chen et al. (18) found that venlafaxine and paroxetine have different immunomodulatory properties in the treatment of patients with MDD. The present finding of the effects of antidepressants on normalizing the serum TNF-α levels in patients with MDD adds to the growing body of literature that suggests that TNF-α may not only be capable of causing depression, but that it also has a role in the modulation of emotional processes.

Consequently, our results demonstrated that serum TNF-α levels at baseline were significantly lower in the AS group than in the AIS group, which is consistent with previous studies (24, 28). Meanwhile, we also found that the higher the baseline serum TNF-α levels, the lesser the improvement in depressive symptoms in this sample. Together with the decreasing TNF-α levels after an effective treatment, this finding has important clinical implications for the novel treatment of patients with MDD, particularly treatment-resistant patients. The existing evidences show that TNF-α blockers are effective in the treatment of chronic inflammatory disorders, such as psoriasis. A recent study has shown that TNF-α blockers were also effective in decreasing depressive symptoms associated with psoriasis (30). In view of the association pattern between TNF-α levels and treatment improvement, it should be verified whether inhibiting the TNF-α levels will have a therapeutic potential in patients with MDD.

Interestingly, we found that two subdomains (anxiety/somatization and weight loss) of the depressive symptoms showed significant correlations with TNF-α levels. Those symptoms can be interpreted as somatization, which is also commonly reported in somatic diseases. First, depression is highly related with other inflammatory diseases (such as inflammatory bowel disease) (31), gastrointestinal problems, appetite changes, and aches and pains of a diffuse nature as common features of depression and inflammatory disease. This raises the possibility that TNF-α, which is activated in several somatic diseases and leads to somatization, may also be involved in the development of depression (3).

Our study has several limitations that should be noted. First, the premise of this study lies in the nature of the assessments conducted in real clinical practice. However, the raters were not informed that the aim of this study was to explore the changes in the serum TNF-α levels before and after treatment. The naturalistic aspect would realistically reflect the changes in the TNF-α levels of these patients in an unbiased fashion. Another limitation is that psychotic symptoms and cognitive impairments were not assessed in this study. The current study relied only on the assessment of depressive symptoms, which is inadequate. In addition, there was a gender difference between the patients and healthy controls. In order to ensure that the TNF-α tests are performed using the same batch of human TNF-α ELISA kit, the healthy controls were recruited at the same time as the patients with MDD. Besides, we did not investigate the body mass index, smoking status, and C-reactive protein; all those variables are important parameters related to inflammation. Finally, this study was limited to small sample size and only the serum TNF-α level was included; other inflammatory and anti-inflammatory cytokines were not assessed due to limited resources.

## Conclusion

In summary, our results revealed that the serum TNF-α levels were higher in patients with MDD than in healthy controls. Following the antidepressant treatment, the TNF-α levels were significantly decreased and comparable to those in the healthy controls. The baseline TNF-α level was correlated with the improvement in depressive symptoms, particularly the symptoms of anxiety/somatization and weight loss. Our findings indicate that TNF-α may be involved in the pathophysiology of depressive symptoms and that it has a predicting value for the treatment response in patients with depressive mood state.

## Data Availability Statement

The datasets generated for this study are available on request to the corresponding author.

## Ethics Statement

The studies involving human participants were reviewed and approved by The Research Ethics Committee of the Nanhui Pudong New Area Mental Health Center of Shanghai. The patients/participants provided their written informed consent to participate in this study.

## Author Contributions

TZ, LY, LP, JW, and MQ conceptualized the study, wrote the first draft of the manuscript, and conducted the statistical analyses. WS and CG helped in the design of the study and edited the manuscript. LH, LC, XT, YH, and LX interviewed the participants and collected and organized the primary data. YW managed the literature searches and statistical analyses and edited the manuscript. TZ and JW designed the study and provided supervision in the implementation of the study. All authors have approved the final manuscript.

## Conflict of Interest

The authors declare that the research was conducted in the absence of any commercial or financial relationships that could be construed as a potential conflict of interest.
